# Loss of TGME49_227100 (Glutaredoxin 5) Disrupts Oocyst Formation and Sporulation in *Toxoplasma gondii*

**DOI:** 10.3390/pathogens15020150

**Published:** 2026-01-30

**Authors:** Fujie Xie, Yuehua Xie, Yilin Yang, Chenxi Zhao, Jingxia Suo, Zhenzhao Zhang, Ruiying Liang, Xinming Tang, Xianyong Liu

**Affiliations:** 1 State Key Laboratory of Veterinary Public Health and Safety, Key Laboratory of Animal Epidemiology and Zoonosis of Ministry of Agriculture, National Animal Protozoa Laboratory and College of Veterinary Medicine, China Agricultural University, Beijing 100193, China; 18236911245@163.com (F.X.); xyh18568331350@163.com (Y.X.); yangyilinszn@163.com (Y.Y.); zcx1800@126.com (C.Z.); suojingxia415@126.com (J.S.); zhangzhenzhao2022@163.com (Z.Z.); 2 Key Laboratory of Animal Biosafety Risk Prevention and Control (North) of MARA, Institute of Animal Science, Chinese Academy of Agricultural Sciences, Beijing 100193, China; liangruiying@caas.cn

**Keywords:** *Toxoplasma gondii*, Grx5, oocyst, sporulation

## Abstract

Oocysts of *Toxoplasma gondii* exhibit remarkable resistance to environmental stressors and most conventional disinfectants. Despite its ability to infect a wide variety of host species, sexual reproduction and oocyst formation occur exclusively within felid definitive hosts. Despite the epidemiological significance of oocyst-mediated transmission, the molecular mechanisms governing oocyst production and sporulation remain incompletely understood. Glutaredoxin, serving as a central regulator of cellular redox homeostasis and multiple vital cellular processes in cells, is a potential regulator for oocyst sporulation. Here, we investigated the role of TGME49_227100 (glutaredoxin 5, Grx5) in the *T. gondii* Pru strain-a type II strain capable of oocyst formation, with a particular focus on its functions during oocyst formation and sporulation. We found that Grx5-knockout tachyzoites exhibited no defects in growth or virulence. Neither in vitro nor in vivo tachyzoite-to-bradyzoite differentiation was affected compared to wild-type parasites. Notably, Grx5 deletion significantly reduced oocyst production in cats by approximately 70%. Additionally, the collected oocysts showed a 50% decrease in sporulation rate. These results indicate that Grx5 plays a predominant role within feline host and the external environmental stage of sporulation, which of these is likely to provide a crucial molecular target for developing a transmission-blocking vaccine.

## 1. Introduction

*Toxoplasma gondii* is a globally prevalent zoonotic apicomplexan parasite capable of infecting a broad range of warm-blooded hosts, including humans [1]. Its life cycle alternates between asexual replication in intermediate hosts and sexual reproduction in its definitive feline hosts [2]. Following sexual reproduction in the feline intestinal epithelium, unsporulated oocysts are shed into the environment, where they undergo sporulation within 1–5 days to become infectious. Sporulated oocysts represent the major source of environmental transmission to intermediate hosts [3]. After ingestion, sporozoites differentiate into tachyzoites, disseminate throughout the host, and eventually establish chronic infection in the form of tissue cysts, primarily in neural and muscular tissues. In addition, consumption of undercooked meat harboring tissue cysts constitutes another important route of human infection.

Oocyst sporulation is a highly regulated developmental process that enables *T. gondii* to survive outside the host and persist under adverse environmental conditions. Mature oocysts are remarkably resistant to physical and chemical stressors, including strong acids, chlorine-based disinfectants, ozone, detergents, and ultraviolet irradiation [4,5]. This resistance is largely attributed to the unique structure and composition of the oocyst wall, which consists of an outer lipid-rich layer that limits permeability to water-soluble agents and an inner layer enriched in cysteine-rich oocyst wall proteins, tyrosine-rich proteins responsible for autofluorescence, and β-1,3-glucan fibers that together confer mechanical strength and stability [6,7,8,9,10]. In addition, stage-specific expression of stress-associated proteins, such as Late Embryogenesis Abundant proteins, has been implicated in enhancing oocyst tolerance to environmental insults during sporulation [11].

Glutaredoxins are conserved redox-regulatory proteins that play essential roles in maintaining cellular redox homeostasis and supporting DNA synthesis through ribonucleotide reductase activity [12,13]. Recent transcriptomic and proteomic analyses have revealed pronounced stage-specific regulation of redox-related genes during oocyst development, among which glutaredoxin 5 (Grx5) is notably upregulated in sporulated oocysts [14,15]. However, despite its distinctive expression profile, the functional contribution of Grx5 to parasite development, particularly during oocyst formation and sporulation, remains unclear.

In this study, we investigated the role of Grx5 in *T. gondii* by generating a Grx5-deficient strain and systematically evaluating its effects across different developmental stages. Our results demonstrate that deletion of Grx5 does not impair parasite replication in vitro or development in intermediate hosts but significantly affects oocyst formation in definitive host and sporulation, highlighting a stage-specific role of Grx5 in the environmental transmission of *T. gondii*.

## 2. Materials and Methods

### 2.1. Parasites, Cell Culture and Animals

The Type II Pru Beijing strain is preserved by the laboratory. Since previous study reported that bradyzoites isolated from cysts can rapidly replicate in primary mouse astrocyte cultures, this approach may compensate for the limitations of primary mature bradyzoites, whose developmental capacity is influenced by host cell type [16]. Therefore, primary murine astrocytes (PMNA) were used to replace standard HFF cells in this study. Brain tissues were collected from 2–3-day-old suckling mice and homogenized by repeatedly passing through a needle using a 5 mL syringe for three cycles. Following filtration through a sterile 40 μm cell strainer, the homogenate was washed twice with pre-cooled PBS and then centrifuged at 2000 rpm for 10 min at 4 °C. For the final step, cells were resuspended and cultured in Dulbecco’s modified Eagle’s medium (DMEM, Macgene, Rockville, MD, USA) containing 10% fetal bovine serum (FBS, Sigma-Aldrich, Saint Louis, MO, USA), cultured under 37 °C with 5% CO_2_. Female CD-1 mice (2–3 day old or 6–8 weeks old) were obtained from Beijing HFK Bioscience Co., Ltd. (Beijing, China) Nine toxoplasma-negative cats were supplied by Beijing Marshall Biotechnology Co., Ltd. (Beijing, China).

### 2.2. Generation and Validation of Deleted or Complementary Parasite Strains

The ToxoDB database (https://toxodb.org (accessed on 25 December 2023)) served as the source for the genome information of TGME49_227100 (glutaredoxin 5, *Grx5*). To construct the knockout strain, we used 800–1100 bp homologous arms from *Grx5* and replaced the target gene with chloramphenicol (CAT) and red fluorescent protein genes. The plasmid pUPRT-Tub-CAT-mCherry-UPRT with the homologous arms of *Grx5*, co-transfection was performed using the specific sgRNA CRISPR/Cas9 plasmid along with the PCR products into freshly collected tachyzoites using program U-033 of Lonza instructions. Incubate the flasks at 37 °C without drug selection to allow the parasites to recover. Twenty-four hours after the recovery period, add fresh astrocyte media containing 40 μg/mL chloramphenicol to the flasks. After PCR screening of the positive population, mice were intraperitoneally injected with the progeny tachyzoites to induce cyst formation. Twenty-five days later, brain cysts were collected and diluted for individual screening of single fluorescent cyst in 96-well plates. Those selected single cysts were then reinoculated into mice. After another 25 days, brain cysts were harvested, and genomic DNA was extracted for PCR confirmation.

To construct the complementary strain, we used 800–1100 bp homologous arms of *Grx5* and replaced the target gene with dihydrofolate reductase (DHFR) and green fluorescent protein, we replaced the homologous arms in pUPRT-Gra2-DHFR-EGFP-UPRT with the homologous arms of TGME49_227100, co-transfected the sgRNA and PCR products into knockout strain, followed by selection with 0.5 µM pyrimethamine, the clone strain was screened as above. Table 1 lists the primers and plasmids employed in this study.

### 2.3. Immunofluorescence Assays

PMNA cells were cultured on coverslips for 7 days prior to inoculation with *Toxoplasma*. The coverslips were fixed with 4% formaldehyde for 30 min, permeabilized with 1% Triton X-100 for 30 min, and blocked with 3% BSA for 30 min. All primary and secondary antibody incubations were performed for 1 h in a humidified chamber by inverting the coverslips onto 50 μL of antibody dilutions prepared in blocking buffer on Parafilm. Three washes with PBS were carried out after each step. The coverslips were mounted with 5 μL of ProLong Diamond Antifade Mountant (Thermo Fisher, Waltham, MA, USA) and allowed to set for 30 min at 37 °C or overnight at room temperature. Primary antibodies and dyes used included: rabbit anti-GAP45 antibody (1:500 dilution), mouse anti-SAG1 antibody (1:500 dilution), and Hoechst 33,258 nuclear dye (1:2000 dilution). Secondary antibodies conjugated with Alexa Fluor 488, 594, or 647 (Thermo Fisher) were applied at a dilution of 1:200.

### 2.4. Invasion Assay

PMNA monolayers cultured in 12-well plates were infected with tachyzoites of *T. gondii* type II Pru strain, including the wild type, Grx5 knockout, and Grx5 complementary strains (2  ×  10^6^ tachyzoites/well), and incubated at 37 °C under 5% CO_2_ for 4 h. Following medium removal, coverslips were fixed with 4% formaldehyde and blocked with 3% BSA, each for 30 min. For surface staining, samples were incubated with mouse anti-SAG1 antibody and then with Alexa Fluor 594-conjugated goat anti-mouse secondary antibody, both for 1 h at 37 °C. After washing with PBS three times and permeabilizing with 1% Triton X-100 for 30 min, intracellular staining was achieved by successive 1 h incubations at 37 °C with rabbit anti-GAP45 antibody and Alexa Fluor 488-conjugated goat anti-rabbit secondary antibody.

Non-invaded tachyzoites exhibited red fluorescence, while both intracellular and extracellular tachyzoites displayed green fluorescence [17].

### 2.5. Replication Assay

PMNA monolayers on coverslips were inoculated with ~10^5^ freshly harvested parasites and cultured for 4 h. Prior to a 48 h incubation, non-invaded parasites were removed by triple PBS washing. The monolayers were then fixed with 4% PFA, stained with anti-GAP45 antibodies, and parasite numbers per vacuole were quantified (100 vacuoles/condition) in three independent experiments.

### 2.6. In Vitro Stage Differentiation Assay

Under neutral culture conditions (DMEM, 2% FBS, pH 7.4), PMNA monolayers were infected with purified tachyzoites for 4 h. The medium was replaced with alkaline RPMI-HEPES medium containing 2% FBS (pH 8.2). Cultures were maintained at 37 °C under ambient CO_2_ levels (~0.03% CO_2_), with daily replacement of the alkaline medium to sustain a high alkaline pH. Parasite cyst wall were labeled with FITC-conjugated Dolichos biflorus lectin as previously described [18], and the parasites were visualized by staining using anti-GAP45 antibody. Cyst development was assessed by calculating the percentage of DBL-positive PV, with at least 100 PVs examined across three independent biological replicates.

### 2.7. Virulence Test and Brain Cysts Counting in Mice

Parasites were harvested as previously described, counted using a hemocytometer, and diluted with PBS. Eight-week-old female CD1 mice were randomly assigned to groups receiving an intraperitoneal injection of 2 × 10^3^ freshly egressed tachyzoites of either the WT, Grx5 knockout, or Grx5 complementary strain (*n* = 6 mice per condition). With daily monitoring, all mice were euthanized once they reached humane endpoints (approximately 4 weeks post-inoculation). Brain tissues were dissected into 2 mL of PBS, homogenized by repeated extrusion through a 20-gauge needle, and subjected to DBL staining. A 15 μL aliquot of the stained homogenate was examined under a fluorescence microscope [19]. Cyst burden was calculated based on the mean of three samples, and the total brain cyst load was determined according to the total volume of the brain homogenate and the average count per 15 μL.

### 2.8. Cat Infection, Oocyst Collection and Sporulation

For oocyst production, *T*. *gondii*-naive kittens (10–12 weeks old) were used in this study, with three cats randomly infected per strain. Briefly, *T. gondii*-infected mouse brain tissue was homogenized using a syringe and administered to the cats by placing the homogenate at the back of the tongue. Feces from each cat were collected daily following feeding with infected mouse brain tissue and examined for *Toxoplasma gondii* oocysts. Screening and harvesting of *T. gondii* oocysts were performed between 3 and 21 days post-infection according to procedures described previously [2]. Oocysts were collected using a flotation method with a sucrose solution (specific gravity ≥ 1.15). The concentrated oocyst pellets were resuspended in an aqueous solution containing 2% H_2_SO_4_ and aerated on a shaker for 14 days at room temperature (20–22 °C) to allow sporulation. Oocysts were counted using a disposable hemocytometer. The total number of oocysts shed by each cat was calculated based on the total count, dilution factor, and total volume.

### 2.9. Statistical Analysis

Statistical analyses were performed using GraphPad Prism version 9 for Windows (9.5.1, GraphPad Software). Data are presented as the mean ± standard deviation (SD). The figure legends contain specifics regarding observations, biological replicates, error bars, and the statistical tests employed, with statistical significance set at *p* < 0.05. All microscopy images shown are representative of findings from a minimum of two independent experiments, all of which produced comparable outcomes.

## 3. Results

### 3.1. Deletion of Grx5 Does Not Impair the Phenotypic Traits of T. gondii Tachyzoites

**Figure 1:**
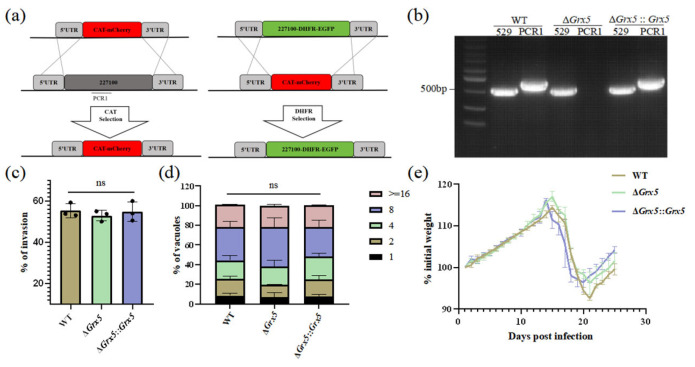
Deletion of Grx5 does not impair the phenotypes of *Toxoplasma gondii* tachyzoites. (**a**) Schematic of the Grx 5 knockout and complementary strategies. (**b**) PCR verification of successful ablation and complementation of Grx 5. Expected product sizes: Rep 529 (529): 0.529 kb. Grx 5 (PCR1): 0.720 kb. (**c**) Invasion assays of parasites. The WT, Δ*Grx 5* and Δ*Grx 5*::*Grx 5* strains showed no significant difference in invasion. Data represent the mean ± SD for three independent experiments, analyzed by two-tailed, unpaired *t* test, and the indicated strains were compared with Pru strain. ns., not significant. (**d**) Growth assays of parasites. The WT, Δ*Grx 5* and Δ*Grx 5*::*Grx 5* strains showed no significant difference in growth. Data are expressed as means ± SD for three independent experiments, analyzed by two-way ANOVA with Tukey multiple comparison test, and the indicated strains were compared with Pru strain. ns., not significant. (**e**) Mean normalized weights of animals in each group. Graph represents mean ± SEM for all surviving animals at a given time point. No mice died during the process (*n =* 6).

To investigate the biological function of Grx5, knocked out this gene was performed in *T. gondii* type II Pru strain using CRISPR-Cas9 technology. For Grx5 knockout, the coding region of Grx5 was replaced by fused coding sequences of CAT and mCherry. While the fragment 5HR-Grx 5-DHFR-EGFP-3HR was used to construct complementary strain (Figure 1a). The targeted *Grx5* gene deletion was confirmed by PCR, which amplified a ~700 bp band in the complemented strain but not in the KO strain (Figure 1b). To investigate the impact of Grx5 deletion on the in vitro growth of *T. gondii*, the invasion assay and replication assay were performed. PMNA monolayers in 12-well plates were infected with WT, Grx5 knockout, and Grx5 complementary strains. Quantification of tachyzoites within PVs by fluorescence microscopy at 4 and 48 h post-infection (hpi) revealed that Grx5 deletion did not significantly affect parasite invasion or proliferation compared to the WT strain (Figure 1c,d). Grx5 deletion did not significantly affect virulence, as demonstrated in a mouse model infected via intraperitoneal injection with freshly egressed tachyzoites (Figure 1e). No mortality was observed in mice of the three groups.

### 3.2. Deletion of Grx5 Does Not Affect Tachyzoite–Bradyzoite Differentiation In Vitro and In Vivo

**Figure 2:**
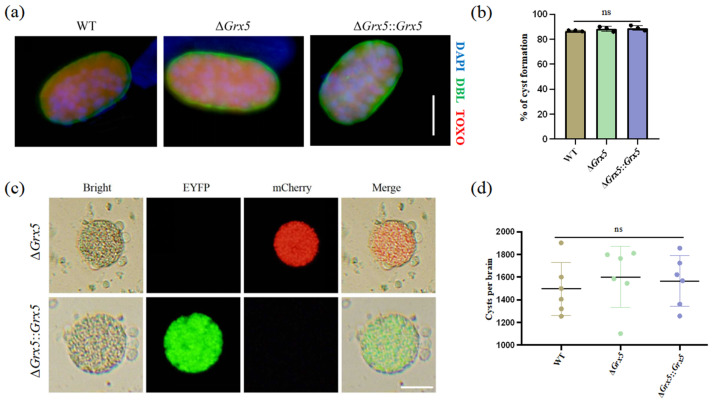
Deletion of Grx5 does not affect tachyzoite–bradyzoite differentiation in vitro and in vivo. (**a**) Representative pictures of cyst of each strain produced in vitro as assessed by DBL staining. Scale bar, 10 µm. (**b**) Grx5 do not effect bradyzoite differentiation in vitro across the parasite strains in bradyzoite condition. Data represent the mean  ±  SD for three independent experiments. Statistical analysis was performed by two-tailed, unpaired *t* test, and the indicated strains were compared with Pru strain. ns., not significant. (**c**) The representative images of brain cysts of knockout strain and the complemented strain. The result showed that the knockout strain exhibits red fluorescence, while in the complemented strain, the red fluorescence is replaced by green fluorescence. Scale bar, 20 µm. (**d**) Brain cyst burden of mice after 4 weeks of infection. Cysts per brain were estimated from counting three blinded replicates, mean is plotted for each group. Data represent the mean  ±  SD for three independent experiments. Statistical analysis was performed by two-tailed, unpaired *t* test, and the indicated strains were compared with Pru strain; ns., not significant.

We also tested the ability of Grx5 knockout strain to differentiate to bradyzoites in vitro under conditions of pH 8.2 stress, as assessed by staining with DBA. It was observed that the cyst formation of the knockout strain was unaffected (Figure 2a). The transformation rates were similar between the WT strain, the knockout strain and the complementary strain (Figure 2b). To assess the impact of Grx5 on cyst development in vivo, the brain cyst burden was quantified in infected mice. Results showed that Grx5 deletion had no significant effect on cysts formation (Figure 2c,d).

### 3.3. Deletion of Grx5 Significantly Reduced Oocyst Production and Sporulation Rate

**Figure 3:**
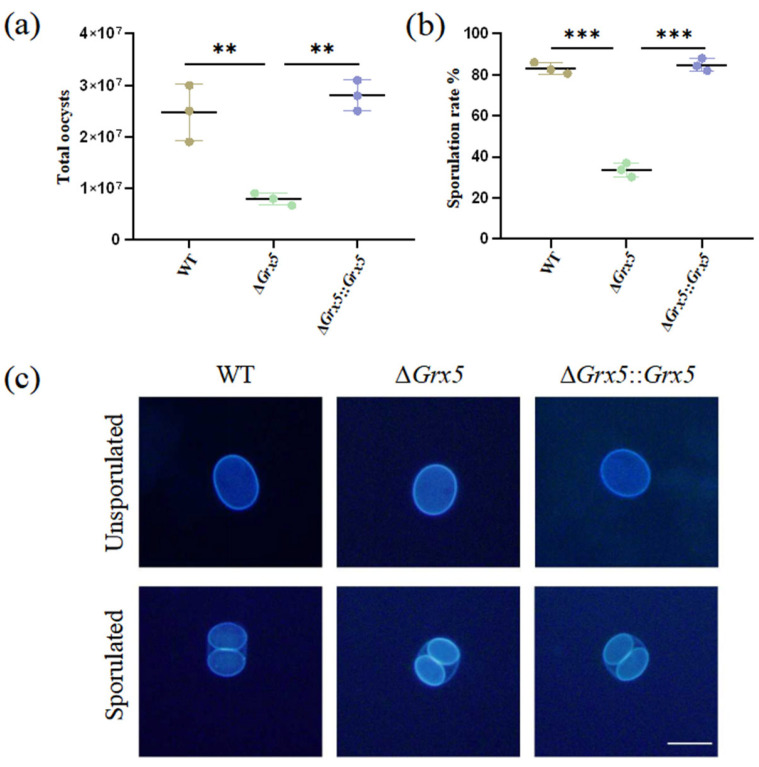
Deletion of Grx5 significantly reduces oocyst production and sporulation. (**a**) Yields of oocysts shed from infected cats. The yield of knockout strain as a whole were significantly reduced relative to the wild-type. Data represent the mean ± SD for three independent experiments. Statistical analysis was performed by two-tailed, unpaired *t* test, and the indicated strains were compared with Pru strain. **, *p* < 0.01. (**b**) The sporulation rate of shed oocysts shows a significant defect in knockout strain. Data represent the mean  ±  SD for three independent experiments. Statistical analysis was performed by two-tailed, unpaired *t* test, and the indicated strains were compared with Pru strain. ***, *p* < 0.001. (**c**) Representative fluorescence microscope images of dityrosine autofluorescence in sporulated and unsporulated oocysts of WT, Δ*Grx 5* and Δ*Grx 5*::*Grx 5* strains. Scale bar, 10 µm.

To study the role of Grx5 during the feline stage and environment stage, tissue cysts contained in mouse brain homogenate were fed orally to cats and oocyst shedding was monitored. Cats commenced to shed oocysts within the first week. Infection with the WT strain consistently yielded around 2.5 × 10^7^ total oocysts during the oocyst shedding period, but the Grx5 deletion strain was markedly reduced, compared to the WT group, with an average value of 8 × 10^6^ (Figure 3a). After the sporulation of oocysts in potassium dichromate solution, the Grx5 deletion strain exhibited a sporulation rate of merely ~30%, markedly reduced compared to the value of ~80% in the WT group (Figure 3b). Although the deletion strain showed defects in the extent of sporulation, while the sporulated oocyst was normal. Interestingly, the fluorescence of the inner sporocyst walls are much stronger than the oocyst wall under UV illumination (Figure 3c).

## 4. Discussion

Our findings demonstrate that the deletion of grx5 does not affect the development of tachyzoites and the differentiation of tachyzoite into bradyzoite, but does impair oocyst shedding and sporogony, underscoring the essential role of this gene in parasite transmission and developmental competence.

The glutathione system serves as a central regulator of antioxidant defense, anti-apoptotic processes, and other biological functions in the body [20,21]. In *Trypanosoma brucei*, cytoplasmic 2-C-GRX1 regulates thermotolerance, while 2-C-GRX2 is essential only in the insect procyclic stage [22,23]. Conversely, in *Neospora caninum*, NcGRX1 and NcGRX5 play a critical role in growth, while NcGRX5 specifically interacting with mitochondrial ISCS and ISCU1 to regulate energy metabolism [24,25,26]. Grx1 is essential for oxidative stress resistance, in vitro growth, and mouse pathogenicity in *T. gondii*, dependent on its catalytic CGFS motif [27]. In our results, we found Grx5 impact the oocyst production and sporulation, which emphasizes that glutaredoxin proteins exhibit distinct and stage-specific functions across parasitic protozoa.

The most plausible explanation for the attenuated phenotype is that the loss of Grx5 disrupts the biogenesis of Fe-S clusters, which are indispensable cofactors for a wide array of proteins [28,29]. This disruption likely impairs the parasite at multiple levels during its development within the feline intestinal epithelium. An impairment here would stall the rapid nuclear divisions required for the formation of meronts and gamonts, effectively reducing the foundational population of parasites available for subsequent gamete formation. This aligns with phenotypes observed in mutants of other metabolic genes (e.g., AAHs), where a general fitness cost in the feline intestine leads to reduced oocyst output [30]. While a general fitness defect is a plausible mechanism for the reduced oocyst output in the Grx5 knockout, a more specific role in late sexual development (e.g., macrogamete formation, fertilization, or oocyst wall biogenesis) cannot be excluded. This distinguishes it from mutants like T-263, which are blocked specifically at zygote formation [31]. However, the proposed model—that Grx5 deficiency impairs intestinal proliferation—remains speculative due to experimental limitations, such as the lack of direct histopathological evidence from infected feline intestine.

The reduced oocyst sporulation rate following Grx5 deletion critically impairs the parasite’s environmental transmission. This likely stems from an energy and biosynthetic collapse during this highly demanding process, preventing the completion of sporozoite development. This distinct phenotype, unlike the reduced shedding seen in mutants like AAHs [30], highlights a specific vulnerability in the extrinsic phase. Future work should directly assess the viability and infectivity of any sporozoites that do form, and analyze the transcriptomic and metabolic profile of wild-type versus mutant oocysts during sporulation to pinpoint the exact biochemical block (e.g., by measuring respiration rates or lipid precursor levels).

In summary, loss of Grx5 markedly compromises the ability of *T. gondii* to form oocysts and undergo sporulation. This phenotype establishes a dual transmission barrier, reducing both oocyst quantity and quality (infectivity). A mutant with fewer, poorly sporulating oocysts exhibits a doubly attenuated phenotype, significantly enhancing the potential of Grx5 as a high-value target for blocking transmission by attacking two sequential points in the parasite’s life cycle.

## 5. Conclusions

This study identifies the glutaredoxin Grx5 as a stage-specific regulator critical for *Toxoplasma gondii* transmission, essential for high-yield oocyst production in the definitive host and efficient sporulation in the environment, while dispensable for asexual stages. The dual phenotype likely stems from Grx5’s role in Fe-S cluster biogenesis, crucial for the metabolic demands of sexual development. Consequently, Grx5 represents a high-value target for transmission-blocking strategies, such as live-attenuated vaccines, aimed at preventing environmental contamination and zoonotic spread.

## Figures and Tables

**Table 1:** The primers used in this study for constructing plasmids and identifying the parasite strains.

Primers	Sequence
Grx 5 5′-F	TATAGGGCGAATTGGGTACCGAGTAACTGTACCGGCTAAC
Grx 5 5′-R	CGATACCGTCGAGGGGGGGCCCTTGAAAAGAGGAAGGCA
Grx 5 3′-F	TAGAGCGGCCGCCACCGCGGGCAGGTCTATCTGCTTCAA
Grx 5 3′-R	GGCGACGCAGGTGCTTTA
CAT-mCherry-F	GGCCCCCCCGACGGTATC
CAT-mCherry-R	CCGCGGTGGCGGCCGCTCTA
PCR 1-F	CGTCATCAAAAGCCACAAGG
PCR 1-R	TGTATCTCGGCCCTTTACTG
REP529-F	CGCTGCAGGGAGGAAGACGAAAGTTG
REP529-R	CGCTGCAGACACAGTGCATCTGGATT
Grx 5 DNA-F	ATGTCGGACAAGTCGAAATG
Grx 5 DNA-R	TAATAAAGCACCTGCATCCTTGCA
DHFR-EYFP-F	TCCTGCACTCGACTTGACGAGG
DHFR-EYFP-R	ACCGCTTTCTCAACAGGAAA

## Data Availability

The datasets supporting the conclusions of this article are available from the corresponding author on request.
